# Structure of ^3^He

**DOI:** 10.1038/s41598-021-98416-8

**Published:** 2021-09-23

**Authors:** P. D. Morley

**Affiliations:** Blue Ridge Scientific LLC, Front Royal, VA 22630 USA

**Keywords:** Experimental nuclear physics, Physics, Nuclear physics

## Abstract

Using electron scattering data, the diffraction pattern off $^{3}$He shows it to be an equilateral triangle possessing dihedral D$_{3}$ point group symmetry (PGS). Previous work showed that $^{4}$He is a 3-base pyramid with C$_{3v}$ PGS. $^{6}$Li is predicted to have C$_{2v}$ PGS. As nuclear $A \rightarrow$ large, atomic nuclei enter into the ‘protein folding problem’ with many possible groundstate PGS competing for lowest energy.

High energy elastic electron scattering off nuclei reveal diffraction patterns that characterize the internal nuclear structure. Using the diffraction data of^1,2^, reference^3^ showed that $^{4}$He is a 3-base pyramid, having C$_{3v}$ PGS. This finding is fatal for the nuclear shell model that **posits mean-field theory** and a (1s)$^{4}$ configuration (literally a sphere) for the $\alpha$-particle. In reality, the atomic nucleus is a **true** many-body system, where each nucleon wavefunction depends critically on the position and spin of every neighbor. It can be argued that the many decades-old $^{3}$He scattering data of^1,2^ (recently joined by the data of^4^ which agrees with the original data) is the most enigmatic nuclear scattering data ever obtained. Point group symmetry of small *A* nuclei is the key to the atomic nucleus. There exists Nuclear Physics Laboratories that have a *Mission Statement* to derive the atomic nucleus from Quantum Chromodynamics. After several decades of existence, they have not fulfilled their *Mission Statement*. This paper addresses some of their physics.

The experimenters themselves have compared the $$^{3}$$He data to phenomenological theories and the reader is invited to see their short-comings. None of the published papers conceived that $$ A = 3$$ nuclei have PGS, much less being an equilateral triangle. Reference^5^ introduces three $$^{2}S_{\frac{1}{2}}$$, three $$^{2}P_{\frac{1}{2}}$$, one $$^{4}P_{\frac{1}{2}}$$ and three $$^{4}D_{\frac{1}{2}}$$ states that have specific wavefunctions chosen for their analytical tractability and physical plausiblity. Even so, the author had to exclude a region of configuration space in order to establish a ‘hole’. The author calls this ‘a three-nucleon repulsive core’. Technically, this paper has physics errors because it has cross-terms between the different irreducible representations of $$S_{3}$$. The final paper reviewed here is the calculation^6^ of the $$^{3}$$He form factor in the meson-exchange model. In the authors’ words: ‘The charge form factors show a striking disagreement with experiment: the theoretical momentum transfer at the first minimum is too high and the height of the second maximum is too low.’ In addition, the famous ‘hole’ in the charge distribution for r = 0 is not reproduced. Of course, the meson-exchange model has other issues beyond the $$A = 3$$ system, but they will not be discussed. This concludes the short literature review. Here it is shown that $$^{3}$$He is an equilateral triangle.

We calculate the charged form factor, ${\tilde{F}}_{ch}$, which in one-photon exchange, is

1 $$\begin{aligned} {\tilde{F}}_{ch}(q^{2}) = \int \rho ({\mathbf{r}})\frac{\sin qr}{qr} d^{3}r \end{aligned}$$

The nuclear charge density $\rho ({\mathbf{r}})$ for ^3^He need not be spherically symmetric. The charge density for point nucleons is ($\tau _{3i}$ is the z-component isospin operator for nucleon numbered *i*)

2 $$\begin{aligned} \rho _{pt}({\mathbf{r}}^{\prime }) = \int \Xi ^{*}_{pt}\sum _{i=1}^{3}\frac{1}{2}(1+\tau _{3i})\delta ^{3}({\mathbf{r}}^{\prime }-{\mathbf{r}}_{i}) \Xi _{pt} d^{3}r_{1}\ldots \end{aligned}$$

Since the proton itself has charge density $\rho _{p}(r)$, then $\rho ({\mathbf{r}})$ is the convolution

3 $$\begin{aligned} \rho ({\mathbf{r}}) = \int \rho _{pt}({\mathbf{r}}^{\prime }-{\mathbf{r}})\rho (r^{\prime })d^{3}r^{\prime } \end{aligned}$$

and now

4 $$\begin{aligned} {\tilde{F}}_{ch}(q^{2}) = {\tilde{F}}_{pt}(q^{2})F_{q}(q^{2}) \end{aligned}$$

where ${\tilde{F}}_{pt}(q^{2})$ is the charge form factor using point nucleons and $F_{q}(q^{2})$ is the Fourier transform of $\rho _{p}$ which is the familiar dipole form factor^7^ $(1+q^{2}(.054842 \mathrm{fm}^{2}))^{-2}$. Experimentalists normalize the charge form factor by $F_{ch}={\tilde{F}}_{ch}/Z$ (*Z* = nuclear charge) so the normalized $F_{ch}(0) = 1$; we will call the normalized charge form factor, ‘the charged form factor’.

We now construct $\Xi _{pt}({\mathbf{r}}_{1}, \mathbf{s}_{1}, \mathbf{t}_{1}, \ldots )$ in which we indicate the position, spin and isospin variables. The S = 1/2, T = 1/2 supermultiplet has symmetry group $S_{3}$ irreducible representations (IR), due to the Pauli Principle. Furthermore, as explained below, Quantum Chromodynamics (QCD) requires that groundstate nuclei have PGS. The vertices of the lattice in the center-of-mass system are (*c* is the base)

5 $$\begin{aligned} \mathbf{a}_{1}= & {} (0, 2 \eta , 0) \nonumber \\ \mathbf{a}_{2}= & {} (c/2, - \eta , 0 ) \nonumber \\ \mathbf{a}_{3}= & {} (-c/2, -\eta ,0) \end{aligned}$$

If $\eta = c/(2 \sqrt{3})$, the triangle is equilateral. The orientation of the lattice plane is immaterial, because the point-like charge density depends only on inner products $\mathbf{a}_{i}\cdot \mathbf{a}_{j}$ (due to the fact that the electron beam is not coherent). Using group theory^8^, the $^{3}$He point wavefunction $\Xi _{pt}$ is made up of the three different IR [$\lambda$] of $S_{3}$: the spatial symmetric $\Psi _{S}$ 1D [3], the spatial anti-symmetric $\Psi _{A}$ 1D [1$^{3}$] and the mixed $\Psi _{M}$ 2D [21].

6 $$\begin{aligned} \Xi _{pt}({\mathbf{r}}_{1}, \mathbf{s}_{1}, \mathbf{t}_{1}, \ldots ) = C_{S}\Psi _{S} +C_{M}\Psi _{M}+C_{A}\Psi _{A} \end{aligned}$$

In Eq. (6), the $C_{i}$ are constants. The reason for the decomposition is that the QCD Hamiltonian has effective nucleon **s**$_{i}\cdot$**s**$_{j}$ spin terms which mix the IR of $S_{3}$. For the basic spatial wavefunction, we take the zero phonon harmonic oscillator (H.O.). The harmonic oscillator parameter is the zero point energy (which is related to the Uncertainty Principle) of the nucleon in the atomic nucleus. We define $\phi ({\mathbf{r}}_{1},{\mathbf{r}}_{2},{\mathbf{r}}_{3})$ to be

7 $$\begin{aligned} \phi (123) \equiv \phi ({\mathbf{r}}_{1},{\mathbf{r}}_{2},{\mathbf{r}}_{3}) = C^{3} \prod _{i=1}^{3}exp[-({\mathbf{r}}_{i}-\mathbf{a}_{i})^{2}/ \alpha ^{2}] \end{aligned}$$

where $C = \frac{1}{\alpha ^{3/2}}(\frac{2}{\pi })^{9/4}$ and $\alpha$ is the H.O. length parameter. In Eq. (7), the ‘123’ only reference the spatial variables ${\mathbf{r}}_{1}, {\mathbf{r}}_{2}, {\mathbf{r}}_{3}$. For simplicity of notation, hereafter, we put $\mathbf{a}_{1} \equiv \mathbf{a},\; \mathbf{a}_{2}\equiv {\mathbf{b}}, \; \mathbf{a}_{3}\equiv {\mathbf{c}}$. The spin-isospin wavefunction *ST*(123) is

8 $$\begin{aligned} ST(123)\equiv & {} \frac{1}{2}[\chi (1)\uparrow \chi (2)\downarrow -\chi (2)\uparrow \chi (1)\downarrow ]\chi (3)\uparrow \cdot \nonumber \\&[\tau (1)\uparrow \tau (3)\downarrow -\tau (3)\uparrow \tau (1)\downarrow ]\tau (2)\uparrow \end{aligned}$$

where $\chi (2)\downarrow$ is nucleon 2 spin down, and $\tau (3)\uparrow$ is nucleon 3 isospin up (a proton). We now construct the individual wavefunctions of Eq. (6) by introducing the Young Tableau of Fig. 1 and the idempotent operators ${{\mathcal {A}}}$, ${{\mathcal {S}}}$ which respectively are the antisymmetrizer and symmetrizer. $^{3}$He has positive parity, with *P* the parity operator.

9 $$\begin{aligned} \Psi _{S}= & {} \frac{1}{2}(1+P){{\mathcal {S}}}[\phi (123)]{{\mathcal {A}}} [ST(123)] \nonumber \\ \Psi _{A}= & {} \frac{1}{2}(1+P){{\mathcal {A}}}[\phi (123)]{{\mathcal {S}}}[ST(123)] \nonumber \\ \Psi _{M}= & {} \frac{1}{\sqrt{2}}[\Psi _{1}{\tilde{\Psi }}_{1} - \Psi _{2}{\tilde{\Psi }}_{2}] \end{aligned}$$

**Figure 1:**
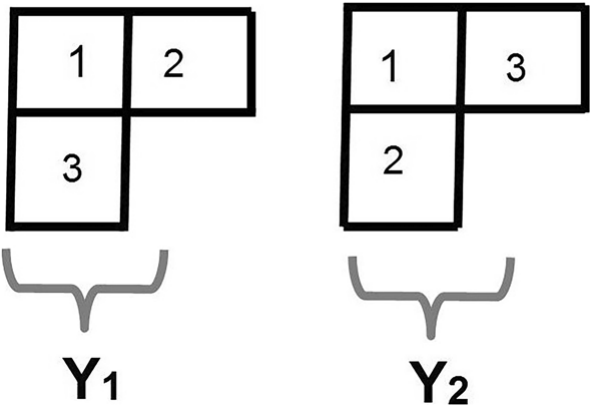
The standard Young Tableau generating the 2D IR of $S_{3}$.

where

10 $$\begin{aligned} \Psi _{1}= & {} \frac{1}{2}(1+P)\frac{Y_{1}}{3}\phi (123) \nonumber \\ {\tilde{\Psi }}_{1}= & {} \frac{Y_{2}}{3}ST(123) \nonumber \\ \Psi _{2}= & {} \frac{1}{2}(1+P)\frac{Y_{2}}{3}(23)\phi (123) \nonumber \\ {\tilde{\Psi }}_{2}= & {} \frac{Y_{1}}{3}(23)ST(123) \end{aligned}$$

It is important for the reader to understand that in the 2D IR of $S_{3}$, a transposition operator such as (23) becomes a matrix in the configuration space spanned by the Young Tableau and the physical wavefunction is the antisymmetric [1$^{3}$] component in the direct product of [21]$\otimes$[21], Eq. (9). There are no cross-terms between the different IR in the calculation of Eq. (2). In conducting the research, one must construct master tables of matrix elements such as $ST(132)\tau _{32}ST(213) = -1/4$ and master tables of expectation values such as $\int \phi (213)P\phi (321)\delta ^{3}({\mathbf{r}}^{\prime }-{\mathbf{r}}_{2})d^{3}r_{1}d^{3}r_{2}d^{3}r_{3} = C^{2}exp\{-[({\mathbf{r}}^{\prime }-\mathbf{a})^{2}+({\mathbf{r}}^{\prime }+{\mathbf{b}})^{2}]/\alpha ^{2}\}exp\{-({\mathbf{b}}+{\mathbf{c}})^{2} / 2 \alpha ^{2}\}exp\{-(\mathbf{a}+{\mathbf{c}})^{2} / 2 \alpha ^{2}\}$. Altogether, there are 36 + 36 + 192 terms in Eq. (2). The results in Fig. 2 are in good agreement with experiment, considering the one-photon scattering approximation and the neglect of neutron scattering.

**Figure 2:**
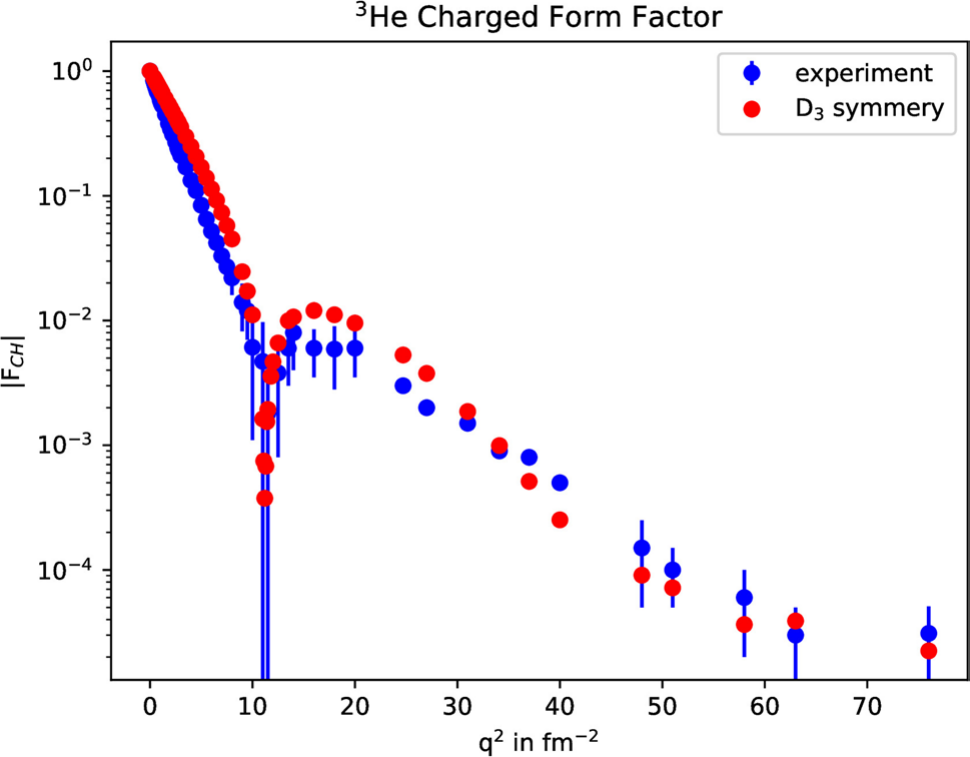
Equilateral triangle scattering of $^{3}$He, using the one-photon approximation and neglecting neutron scattering.

**Figure 3:**
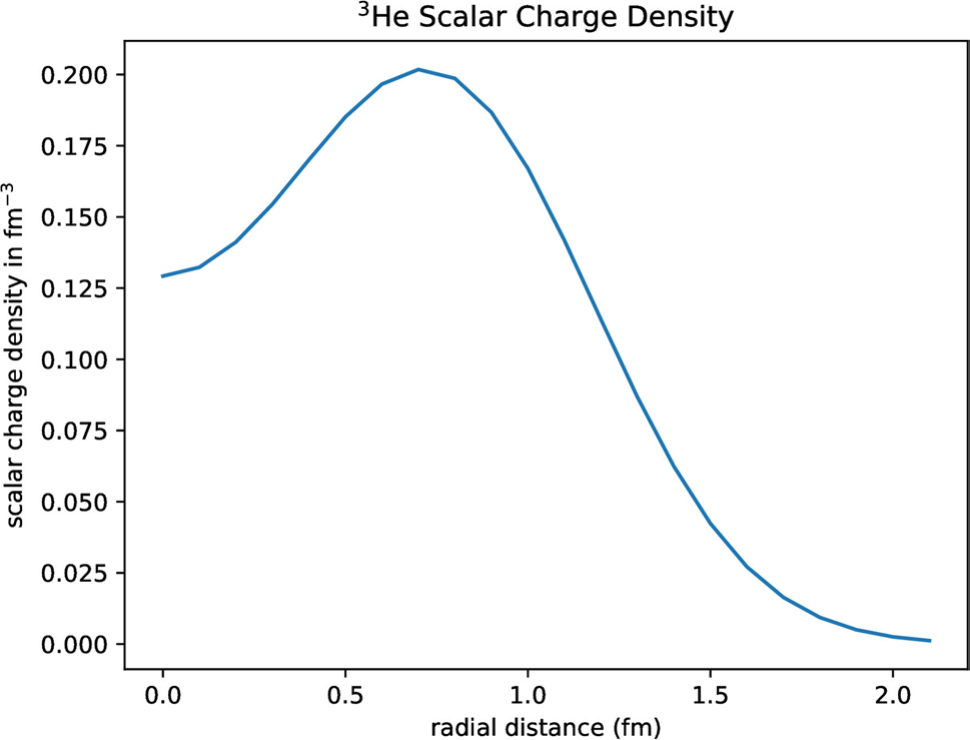
Scalar charge density of $^{3}$He, showing the ‘hole’ at the center.

In Table 1, we give the PGS parameters for $^{3}$He and $^{4}$He. Basically, the extra nucleon in $^{4}$He sits on a with-drawn equilateral triangle base of $^{3}$He. This is understandable because of the existence of a 4-body force in $^{4}$He, discussed below. The radial (scalar) point nucleon nuclear density is

11 $$\begin{aligned} \rho _{pt}(r) = \frac{1}{4\pi }\int \rho _{pt}({\mathbf{r}})d\Omega \end{aligned}$$

which is displayed in Fig. 3. We see $^{3}$He has a ‘hole’ at the center. Finally, we calculate the root-mean-squared $R_{He}$ mass radius of $^{3}$He, which is

12 $$\begin{aligned} R_{He} = \sqrt{<\Xi _{pt}|\sum _{i=1}^{3}r_{i}^{2}|\Xi _{pt}>} \end{aligned}$$

This is done by assembling a master table of expectation values, such as $\int \phi (132)\sum _{i=1}^{3}r^{2}_{i}P\phi (321)d^{3}r_{1}d^{3}r_{2}d^{3}r_{3} = exp\{-(1/2\alpha ^{2})([{\mathbf{b}}+{\mathbf{c}}]^{2} + [\mathbf{a}+{\mathbf{c}}]^{2} + [{\mathbf{b}}+\mathbf{a}]^{2})\}[\frac{9}{4}\alpha ^{2} + (\frac{\mathbf{a}-{\mathbf{c}}}{2})^{2} + (\frac{\mathbf{a}-{\mathbf{b}}}{2})^{2} + (\frac{{\mathbf{b}}-{\mathbf{c}}}{2})^{2}]$. The mass radius is the physical extent of the wavefunction (size of nucleus).

The atomic nucleus is the solution of the N-quark low energy semi-relativistic Hamiltonian. For N=3, reference^9^ solved for the complete $J^{\pi }$ N, $\Delta$ family using the 2-body charmonium potential^10^. (Recently, four-quark matter has been found, and it is anticipated it may have a PGS shape^11^. This further substantiates the Charmonium potential.) Kiefer was able to predict the known N, $\Delta$ $J^{\pi }$ states and all the known photon decay amplitudes for transitions to the nucleon groundstate. Reference^12,13^ solved the N = 6 quark problem and showed that the physical deuteron was due to quark-exchange Feynman diagrams. Reference^14^ showed that the quark-exchange forces give rise to effective nucleon-nucleon potentials. Reference^15^ showed that QCD has 2-, 3-, 4-body quark exchange forces. Finally, reference^16^ was able to concatenate the N-quark Hamiltonian into a nuclear code. This showed that the atomic nucleus groundstate has PGS, while excitations are coherent (keeping the nuclear bonds intact: rotations and vibrations) and incoherent (breaking the nuclear bonds). The saturation of atomic forces is due to the fact that nucleons have only three quarks to exchange: the four-body quark exchange force, due to the gluon 4-body interaction, is the strongest binding mechanism for the atomic nucleus, reference^16^. The nuclear code can be expanded to predict groundstate spins, by noting that the 2-body, 3-body bonds are spin-dependent. For example, the binding energy of the A = 6 nucleus is $E(6) = E_{4} +2E_{3} +4 E_{2}$, where $E_{i}$ are the binding energies of the i-body bonds, so the $E_{2}$ spins cancel, leaving $2E_{3}$-spins ($J^{\pi } = 1^{+}$). Similarly, $E(7) = E_{4}+3E_{3}+4E_{2}$, with the $E_{2}$ spins canceling leaving 3$E_{3}$ spins ($J^{\pi }= \frac{3}{2}^{-}$).

**Table 1:** PGS comparison $^{3}$He with $^{4}$He.

PGS parameters/results	^3^He	^4^He
% Spatial antisymmetric	None discernable	None discernable
% Spatial symmetric	15.99	13.6
% Spatial mixed	84.01	86.4
$\alpha ^{2}$ H.O. (fm^2^)	0.644327	0.644327
3-Base length (fm)		1.341
Equilateral length (fm)	1.61	
Mass radius (fm)	2.023	1.79

The key quantity allowing the nuclear interactions to occur is the overlap of nucleon wavefunctions in the atomic nucleus. There are two radii for the nucleon: the electromagnetic and the mass. For the nucleon in the S = T = 1/2 state, the former radius-squared is $r^{2}_{Q-N}$

13 $$\begin{aligned} r^{2}_{Q-N} = <\frac{1}{2}\frac{1}{2}\left| \sum _{i=1}^{3}\left( \tau _{3i}+\frac{1}{6}\right) r^{2}_{i}\right| \frac{1}{2}\frac{1}{2}>\end{aligned}$$

which is a negative value for the neutron. Physically speaking, the electromagnetic radius measures the internal charge distribution while the mass radius $r_{M}$

14 $$\begin{aligned} r^{2}_{M} = <\frac{1}{2}\frac{1}{2}\left| \sum _{i=1}^{3}r^{2}_{i}\right| \frac{1}{2}\frac{1}{2}>\end{aligned}$$

measures the physical size. In realty, the neutron mass radius and the proton mass radius are nearly identical in value because the gluons in Quantum Chromodynamics do not couple to electric charge. The mass radius of the nucleon is^9^ $\sim 1.38{-}1.40$ fm, showing that the atomic nucleus has overlaping nucleon wavefunctions, allowing QCD color interactions to occur between colorless hadrons. An important experiment that can be conducted is high-energy elastic scattering off $^{6}$Li, which is predicted to have C$_{2v}$ PGS, reference^16^. However, as the nuclear $A \rightarrow$ large, it becomes very difficult to ascertain the geometry of the groundstate wavefunction, the ‘protein folding problem’. For large *A* nuclei, one must consider that the Jahn-Teller effect^17^ may appear, changing the assumed PGS.
